# Protective Effects of Hif2 Inhibitor PT-2385 on a Neurological Disorder Induced by Deficiency of Irp2

**DOI:** 10.3389/fnins.2021.715222

**Published:** 2021-10-05

**Authors:** Jiaqi Shen, Li Xu, Yuxuan Li, Weichen Dong, Jing Cai, Yutong Liu, Hongting Zhao, Tianze Xu, Esther Meyron Holtz, Yanzhong Chang, Tong Qiao, Kuanyu Li

**Affiliations:** ^1^Jiangsu Key Laboratory of Molecular Medicine, Medical School of Nanjing University, Nanjing, China; ^2^Department of Neurology, The Affiliated Jinling Hospital of Nanjing University Medical School, Nanjing, China; ^3^Department of Vascular Surgery, The Affiliated Drum Tower Hospital of Nanjing University Medical School, Nanjing, China; ^4^The Laboratory of Molecular Nutrition, Faculty of Biotechnology and Food Engineering, Technion – Israel Institute of Technology, Haifa, Israel; ^5^College of Life Science, Hebei Normal University, Shijiazhuang, China

**Keywords:** iron regulatory protein 2, hypoxia inducible factor 2α, glycolysis, oxidative phosphorylation, iron sulfur cluster, neurodegeneration

## Abstract

Iron regulatory protein 2 (IRP2) deficiency in mice and humans causes microcytic anemia and neurodegeneration due to functional cellular iron depletion. Our previous *in vitro* data have demonstrated that Irp2 depletion upregulates hypoxia-inducible factor subunits Hif1α and Hif2α expression; inhibition of Hif2α rescues Irp2 ablation-induced mitochondrial dysfunction; and inhibition of Hif1α suppresses the overdose production of lactic acid derived from actively aerobic glycolysis. We wonder whether Hif1α and Hif2α are also elevated *in vivo* and play a similar role in neurological disorder of *Irp2*^–/–^ mice. In this study, we confirmed the upregulation of Hif2α, not Hif1α, in tissues, particularly in the central nervous system including the mainly affected cerebellum and spinal cord of *Irp2*^–/–^ mice. Consistent with this observation, inhibition of Hif2α by PT-2385, not Hif1α by PX-478, prevented neurodegenerative symptoms, which were proved by Purkinje cell arrangement from the shrunken and irregular to the full and regular array. PT-2385 treatment did not only modulate mitochondrial morphology and quality *in vivo* but also suppressed glycolysis. Consequently, the shift of energy metabolism from glycolysis to oxidative phosphorylation (OXPHOS) was reversed. Our results indicate that Irp2 depletion-induced Hif2α is, *in vivo*, in charge of the switch between OXPHOS and glycolysis, suggesting that, for the first time to our knowledge, Hif2α is a clinically potential target in the treatment of IRP2 deficiency-induced neurodegenerative syndrome.

## Introduction

Iron is an indispensable element in mammals. Maintaining proper iron concentration in our body is of great significance because iron, in forms of heme, iron–sulfur cluster (Fe–S), or iron itself as important cofactors, is involved in multiple biochemical pathways, including hemoglobin (HGB) synthesis and mitochondrial respiratory chain (Hentze et al., 2004; Darshan et al., 2010; Ganz and Nemeth, 2012; Rouault, 2013). For this reason, mammals have developed sophisticated mechanisms to maintain proper iron concentration in the body: (1) systemic iron homeostasis is maintained by hepcidin–ferroportin (hepcidin–FPN1, encoded by *HAMP* and *SCL40A1*) axis (Nemeth et al., 2004; Ganz and Nemeth, 2011) and (2) cellular iron homeostasis is mediated by iron regulatory proteins [IRPs, IRP1, and iron regulatory protein 2 (IRP2), also called ACO1 and iron-responsive element-binding protein 2 (IREB2)] through IRP–iron responsive element (IRE) system (Rouault, 2006; Wallander et al., 2006; Muckenthaler et al., 2008; Rouault, 2013). More tissue-specific strategies have also been developed, e.g., ferritinophagy to regulate erythropoiesis (Mancias et al., 2015). These ways function and interplay to fine-tune iron levels in the body (Zhang et al., 2014).

IRP1 and IRP2 are both iron-regulatory RNA binding proteins that regulate the expression of a series of iron-related genes at the post-transcriptional levels (Hentze et al., 2010; Anderson et al., 2012). Under conditions of iron deficiency, the IRE in the target mRNA can be recognized and bound by IRPs, but the consequence of IRP binding depends on the position of the IRE on the mRNA of the target genes. If the IRE is in the 5′-untranslated region (UTR) of the target mRNA, the binding of IRPs may inhibit the translation of the genes, including L- and H-ferritin and FPN1; if the IRE is in the 3′-UTR, the binding of IRPs may stabilize the mRNA, such as that of transferrin receptor 1 (TfR1) (Casey et al., 1988; Müllner et al., 1989) and divalent metal transporter 1 (DMT1) (Tybl et al., 2020). When cellular iron is sufficient, IRP1 binds to a (4Fe–4S) cluster and, therefore, gains aconitase activity and loses the ability to bind IRE, whereas IRP2 is removed by iron and oxygen-mediated proteasome degradation (Salahudeen et al., 2009; Vashisht et al., 2009) to avoid the excessive iron uptake and to promptly store excess intracellular iron and/or export excess iron.

Studies in animal models have shown that IRPs also play an important role in the regulation of systemic iron homeostasis. It has been reported that *Irp2*^–/–^ mice suffer from microcytic anemia (Cooperman et al., 2005; Galy et al., 2005), neurologic defects (LaVaute et al., 2001; Jeong et al., 2011), and diabetes (Santos et al., 2020). The cause of these symptoms is considered to be lack of functional iron in erythroblast progenitors, the cells in central nervous system (CNS), and β cells of *Irp2*^–/–^ mice. The patient with bi-allelic loss-of-function variants in the gene IREB2 leading to an absence of IRP2 also presented neurological and hematological features (Costain et al., 2019), similar, but much more severely, to the observation in *Irp2*^–/–^ mice. The symptoms could be caused by the deficiency of Fe–S biogenesis, which further compromised the mitochondrial quality (Li et al., 2018, 2019).

Recently, we found that mitochondrial dysfunction was closely associated with the reduced expression of a number of genes that are involved in Fe–S biogenesis and mitochondrial respiratory chain (Li et al., 2018). Further investigation revealed that Irp2 may function as a key to switch the metabolism between aerobic glycolysis and oxidative phosphorylation (OXPHOS), mediated by upregulation of hypoxia-inducible factor subunits Hif1α and Hif2α in mouse embryonic fibroblasts (MEFs) (Li et al., 2019). Hif1 and Hif2 are two important transcription factors that can regulate the expression of a series of genes. Active Hif is a heterodimer, composed of an α subunit (Hif1α or Hif2α) and a β subunit (Hif1β, also called ARNT), and can bind hypoxia-responsive element (HRE), which is a very important mechanism in intestinal iron absorption (Mastrogiannaki et al., 2009; Shah et al., 2009) and under other conditions, such as cancer (Keith et al., 2011) and ischemia (Kapitsinou et al., 2014; Barteczek et al., 2017). IRP1 can bind to the IRE in the 5′-UTR of Hif2α mRNA to regulate Hif2α at the post-transcriptional level (Sanchez et al., 2007; Zimmer et al., 2008). In *Irp1*^–/–^ mice, elevated Hif2α upregulates erythropoietin (EPO), causing the mice to develop polycythemia and pulmonary hypertension (Anderson et al., 2013; Ghosh et al., 2013; Wilkinson and Pantopoulos, 2013). Interestingly, in *Irp2*^–/–^ MEFs, we found that Hif1α and Hif2α were both upregulated, which switches the metabolism type from OXPHOS to glycolysis (Li et al., 2019). Inhibition of both Hif1α and Hif2α reversed the energy metabolism.

In this study, we confirmed the elevated Hif2α, not Hif1α, in *Irp2*^–/–^ mice. The upregulated glycolytic pathway-related proteins were also observed and associated with the enhanced glycolysis, while downregulated frataxin (Fxn) and iron–sulfur cluster scaffold protein (IscU), respectively, were observed and associated with deficiency of iron–sulfur clusters. Consequently, the expression of electron transport chain (ETC) subunits was reduced and OXPHOS was weakened. After the inhibition of Hif2α by PT-2385, the energy metabolism was shifted from glycolysis to OXPHOS in *Irp2*^–/–^ mice, the histological and behavioral indicators were restored, and neurodegenerative symptoms were alleviated. Our results indicate that the neurodegenerative disorder induced by the loss of Irp2 is, at least partially, mediated by the upregulated Hif2α.

## Materials and Methods

### Mice

The *Irp2*^+/–^ heterozygous mice were obtained by crossing the purchased *Irp2*^–/–^ mice (purchased from MMRRC at UC Davis, United States, Cat. No. 030490-MU) with wild-type (WT) C57/BL6 mice. Both *Irp2*^–/–^ and WT mice used in the experiment were the descendants of *Irp2*^+/–^ heterozygous mice. The mice were fed a standard rodent pellet diet (200 mg iron/kg) and maintained a constant 12-h light/dark cycle. All procedures were carried out according to the NIH Guide for the Care and Use of Laboratory Animals and were approved by the Animal Experimentation Administration of Nanjing University.

### Experimental Groups and Drug Treatment

A total of 40 mice were divided into four groups: WT mice with vehicle [dimethylsulfoxide (DMSO)] administration (WT DMSO, *n* = 10), *Irp2*^–/–^ mice with DMSO administration (*Irp2*^–/–^ DMSO, *n* = 10), *Irp2*^–/–^ mice with PT-2385 administration (0.4 mg/kg) (*Irp2*^–/–^ PT-2385, *n* = 10), and *Irp2*^–/–^ mice with PX-478 administration (5 mg/kg) (*Irp2*^–/–^ PX-478, *n* = 10).

Both PT-2385 and PX-478 were dissolved in DMSO, diluted with normal saline, and injected intraperitoneally into 6-month-old male *Irp2*^–/–^ mice for 1 month. The injection doses of PT-2385 and PX-478 were 0.4 mg/kg body weight and 5 mg/kg body weight, respectively, and the injection was carried out every other day.

### Behavior Tests

#### Hang Tests

Hang test was performed to assess the grip strength. In the hang test, mice were allowed to grip a wire mesh square that was then inverted. The latency time that mice could hang on to an inverted wire mesh square before falling was measured, and each mouse was tested for three times with an interval of 5 min (*n* = 10).

#### Rotarod Tests

The motor functions of balance and coordination were assessed using an accelerating rotarod (Jiangsu SANS Technology Co., Ltd.). The staying time of mice on the rotating rod (the rotating rod accelerated from 4 to 40 rpm within 5 min) was recorded, and each mouse was tested for three consecutive times (*n* = 10).

### Blood Routine Tests

The red blood cell (RBC), HGB, hematocrit (HCT), and mean corpuscular volume (MCV) were detected by Mindray automatic hematology analyzer (BC-2800vet, Shenzhen, China; *n* = 5).

### Hematoxylin–Eosin Staining

In hematoxylin–eosin (H&E) staining, tissue sections were dealt with the following steps: dewaxed for 10 min in xylene twice, hydrated for 5 min in each 100–50% ethanol gradient buffers, rinsed for 5 min in running water at room temperature, and stained with hematoxylin for 10 min and then in Eosin Y for 10 min. Slides were dehydrated through gradual ethyl alcohol solutions for imaging (*n* = 3).

### Electron Microscopy

The cerebellum and spinal cord were separated in the size of rice grains, placed in a mixed solution of 2% paraformaldehyde and 0.1 M cacodylate for 30 min at room temperature, and then stored at a constant temperature of 4°C (inside a refrigerator). The samples were rinsed once or twice, dehydrated through a series of ethanol from 50 to 100%, and then propylene oxide was used instead of ethanol. The samples were stored in 50% propylene oxide and 50% EPON resin (1:1 mix) for 1 h and then placed in pure EPON. The samples were transferred to fresh EPON in molds or BEEM Embedding Capsules, which were filled carefully to avoid air bubbles, and kept at 60°C for at least 24 h. Samples were observed and photographed by using HT7800 electron microscope at 80 keV, and electron micrographs were commented by Hitachi TEM system (*n* = 3).

### Quantitative Real-Time PCR

Total RNA was isolated with the RNA isolater Total RNA Extraction Reagent (Vazyme, Nanjing, China), and cDNA was obtained by using HiScript^®^ III RT SuperMix for qPCR (+gDNA wiper) (Vazyme, Nanjing, China). qPCR operates by ChamQ^TM^ Universal SYBR^®^ qPCR Master Mix (Vazyme, Nanjing, China). The results were normalized against actin levels. The following primers were used: for actin, forward primer 5′-GCCACTGCCGCATCCTCTTC-3′ and reverse primer 5′-AGCCTCAGGGCATCGGAACC-3′; for EPO, forward primer 5′-AGTTGCCTTCTTGGGACTGA-3′ and reverse primer 5′-GCCACTCCTTCTGTGACTCC-3′; for hepcidin, forward primer 5′-CTCCTGCTTCTCCTCCTTGC-3′ and reverse primer 5′-GCAATGTCTGCCCTGCTTTC-3′; for endothelin-1 (Edn1), forward primer 5′-CCAGGCAGTTAGATGTCAGT-3′ and reverse primer 5′-CCAGCTGCTGATAGATACAC-3′; for lactate dehydrogenase A (LdhA), forward primer 5′-ACTGTGTAACTGCGAACTCC-3′ and reverse primer 5′-CCAC GTAGGTCAAGATATCC-3′; for glucose transporter 1 (Glut1), forward primer 5′-AGGCTTGCTTGTAGAGTGAC-3′ and reverse primer 5′-CAGTGTTATAGCCGAACTGC-3′; and for hexokinase 2 (Hk2), forward primer 5′-TGATCGCCTGCTTATT CACGG-3′ and reverse primer 5′-AACCGCCTGAAATCT CCAGA-3′. The results were normalized against actin levels (*n* = 3).

### Western Blot Analysis

The total protein of each entire tissue was extracted and analyzed (25–35 μg total protein/lane) by 7.5–12.5% SDS-PAGE at 100 V, transferred onto nitrocellulose membrane at 250 mA for 1.5 h, and analyzed by immunoblotting. The information of the primary antibody is as follows: anti-ferritin light chain (cat# 69090), Hif2α (cat# 109616), NcoA4 (cat# ab86707), and SdhB (cat# 178423) from Abcam (Cambridge, MA, United States); anti-TfR (cat# 136800) from Zymed (San Francisco, CA, United States); anti-beta-actin (cat# BM0627) from Boster (Wuhan, China); anti-Hk2 (cat# 22029-1-AP), Glut1 (cat# 21829-1-AP), LdhA (cat# 19987-1-AP), Ndufs1 (cat# 12444-1-AP), and Uqcrfs1 (cat# 18443-1-AP) from Proteintech Group Inc. (Chicago, IN, United States); anti-Hif1α (cat# 14179) from Cell Signaling Technology Inc. (Shanghai, China); anti-ferritin heavy chain (cat# AJ1290b) from ABGENT (San Diego, CA, United States), and anti-Fxn, IscU, Irp1, and Irp2 (polyclonal, self-made, raised from rabbits). All the self-made antibodies were validated in previous studies (Li et al., 2018, 2019). When it is necessary to detect multiple proteins in one blot and the molecular weight of the protein is different, we cut the blotted nitrocellulose membrane according to the molecular weight and then incubate with different antibodies. When the molecular weights are very close, we ran multiple gels with the same prepared total protein samples, transfer them to nitrocellulose membranes, cut them according to molecular weights, and then incubate them with different antibodies. We used Tanon Science and Technology Co., Ltd. (Shanghai, China) ECL-plus reagent to visualize the detected proteins. The intensity of the western blot band was quantified by ImageJ software. Each experiment was repeated at least three times independently, and biological replicates were performed in parallel each time. The average intensity of the bands from replicate samples was first normalized to an internal control (actin) and then normalized to a WT control, with the value set to 1. The final value was the average value from at least three independent experiments (*n* = 3/4).

### Enzyme-Linked Immunosorbent Assay

The serum EPO and interleukin-6 (IL-6) levels were quantified using specific enzyme-linked immunosorbent assay (ELISA) kits according to the manufacturers’ instructions (Invitrogen, *n* = 3).

### Ferrozine Iron Assay

The serum, intestinal, cerebellum, and spinal cord iron contents were detected by the ferrozine iron assay. We took 50 μl of lysate or serum (we took double volume for lysis buffer as control) and added 11 μl concentrated HCl (11.6 M). All the tubes were then placed on a heating block for 20 min at 95°C, centrifuged at the highest speed for 10 min, and then removed very gently from the centrifuge. Very carefully, 45 μl of supernatant was removed. To each tube, 18 μl of ascorbate (75 mM) was added; ascorbate acts as a reductant, moving Fe from 3^+^ state to the 2^+^ state. The tubes were vortex-quick spun and incubated for 2 min, and 18 μl ferrozine (10 mM) was added to each tube. Ascorbate acts as an oxidant, taking Fe from the 2^+^ state to the 3^+^ state. The tubes were incubated for another 2 min. Then, 36 μl of saturate ammonium acetate (NH_4_Ac) was added to each tube and the tubes were incubated for 2 min. Samples were read at 562 nm using a multifunctional fluorescent microplate reader (*n* = 3–5).

### Enzymatic Activities

The activities of complexes I and II were measured according to the manufacturer’s protocols, respectively. Both kits were purchased from Comin Biotechnology Co. (Suzhou, Jiangsu, China; *n* = 5).

### Determination of ATP Content

The levels of ATP in tissues were detected by using an ATP determination kit (Beyotime Biotechnology). The reading is measured by GloMax^TM^ 96-well plate luminescence detector (E6521, *n* = 5).

### Lactic Acid Production

The tissue lysates were collected and assayed according to the lactic acid production detection kit (Nanjing Jiancheng Bioengineering Institute). The assay was detected by a multifunctional fluorescent microplate reader at 530 nm (*n* = 5).

### Statistical Analysis

Student’s *t*-test or one-way analysis of variance (ANOVA) was carried out using GraphPad Prism 8. The measurement was expressed as the mean ± SD; all the experiments were repeated more than three times independently. Significance was considered at *P* < 0.05.

## Results

### Glycolysis-Related Gene Expression Is Enhanced and Oxidative Phosphorylation-Related Gene Expression Is Weakened in the Central Nervous System Tissues of *Irp2*^–/–^ Mice

**FIGURE 1:**
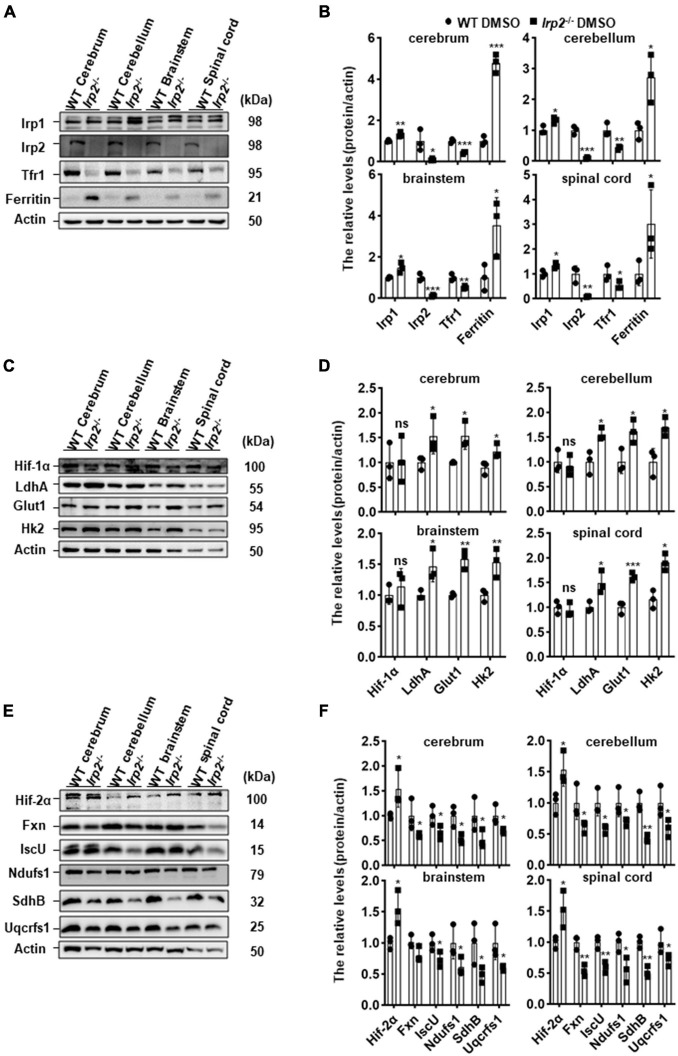
*Irp2* ablation enhances glycolysis-related gene expression and diminishes oxidative phosphorylation (OXPHOS)-related gene expression in the tissues of the CNS, analyzed by western blots. **(A)** Protein levels of iron-related genes (*Irp1*, *Irp2*, *Tfr1*, and ferritin) in central nervous tissues (cerebrum, cerebellum, brainstem, and spinal cord) of wild-type (WT) and *Irp2*^–/–^ mice. **(B)** Quantification of the band intensity for panel **(A)**. **(C)** Protein levels of hypoxia-inducible factor 1α (Hif-1α) and glycolytic pathway-related proteins (Glut1, Hk2, and LdhA) in central nervous tissues (cerebrum, cerebellum, brainstem, and spinal cord) of WT and *Irp2*^–/–^ mice. **(D)** Quantification of the band intensity for panel **(C)**. **(E)** Protein levels of hypoxia-inducible factor 2α (Hif-2α), Fe–S biogenesis-related genes (Fxn and IscU), and mitochondrial complex subunits (Ndufs1 for complex I, SdhB for complex II, and Uqcrfs1 for complex III). **(F)** Quantification of the band intensity for panel **(E)**. Actin was used as a loading control. Values represented as the mean ± SD (*n* = 3), and Student’s *t*-test was used for statistics to evaluate the group differences. In panels **(B,D,F)**, *P < 0.05, **P < 0.01, ***P < 0.001, ^ns^*P* > 0.05, *Irp2*^–/–^ mice vs. WT mice.

Our previous *in vitro* study demonstrated that metabolism switch took place from OXPHOS to glycolysis in *Irp2*^–/–^ MEFs (Li et al., 2019). We wonder if it is the case *in vivo*. First, we detected the expression levels of iron-related proteins in the CNS tissues (cerebrum, cerebellum, brainstem, and spinal cord) of *Irp2*^–/–^ mice. Compared with that in WT mice, ferritin expression was increased while TfR1 was decreased in *Irp2*^–/–^ mice (Figures 1A,B), which was in line with a previous study (Jeong et al., 2011). Next, we detected the expression levels of Hif1α and glycolysis-related proteins and did not find the same elevation of Hif1α as we observed in *Irp2*^–/–^ MEF (Li et al., 2019). However, glycolysis-related proteins, including LdhA, Glut1, and Hk2, were upregulated, compared with that in WT mice (Figures 1C,D), although these genes are also thought to be the members of Hif1 regulon. Then, we detected the expression levels of Hif2α, the Fe–S biogenesis-related proteins (IscU and Fxn), and mitochondrial respiratory complex subunits (Ndufs1, SdhB, and Uqcrfs1). We found that the levels of Hif2α increased by around 50% in general; the levels of IscU and Fxn reduced to 50–70%; and the levels of subunits of complex I (Ndufs1), II (SdhB), and III (Uqcrfs1) also reduced to 50–60% in CNS tissues of *Irp2*^–/–^ mice, compared with WT mice (Figures 1E,F), suggesting a reduction of OXPHOS. Taken together, our results confirmed the biochemical changes *in vivo* related to energy metabolism, OXPHOS, and glycolysis, in the CNS tissues of *Irp2*^–/–^ mice.

### Administration of PT-2385 Significantly Improves the Behavioral Performance and Anemia of *Irp2*^–/–^ Mice

**FIGURE 2:**
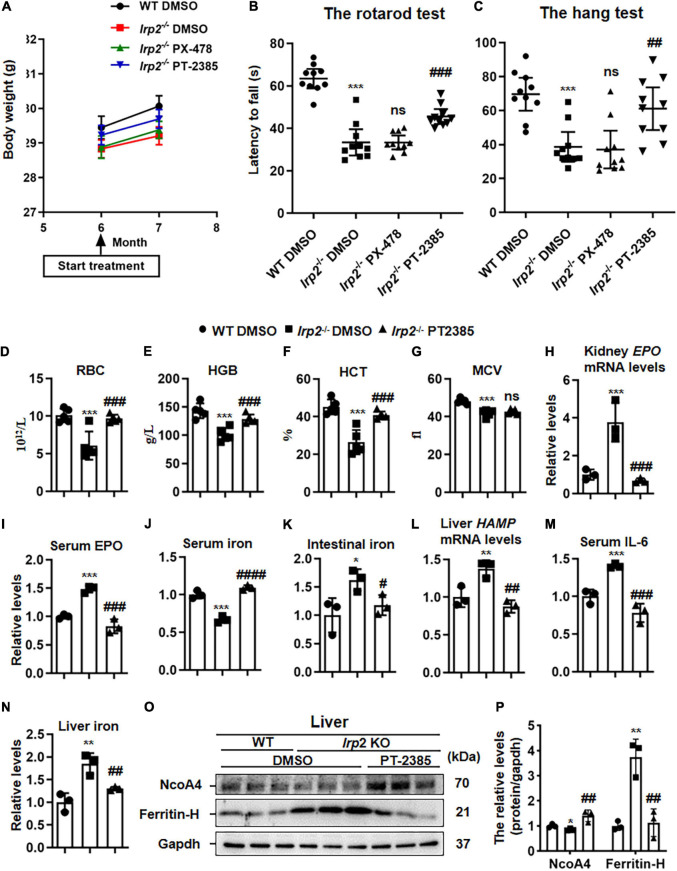
The administration of Hif2α inhibitor PT-2385, not Hif1α inhibitor PX-478, improves the behavioral performance and corrects anemia of *Irp2*^–/–^ mice. Mice were divided into four groups: WT mice with vehicle [dimethylsulfoxide (DMSO)] administration (WT DMSO), *Irp2*^–/–^ mice with DMSO administration (*Irp2*^–/–^ DMSO), *Irp2*^–/–^ mice with PT-2385 administration (*Irp2*^–/–^ PT-2385, 0.4 mg/kg), and *Irp2*^–/–^ mice with PX-478 administration (*Irp2*^–/–^ PX-478, 5 mg/kg). **(A)** The weight of mice before and after the drug administration. **(B,C)** The behavioral tests of mice, including the rotarod tests **(B)** and the hang tests **(C)**; values are represented as the mean ± SD (*n* = 10). **(D)** The number of the red blood cells (RBCs). **(E)** Hemoglobin concentration (HGB). **(F)** Hematocrit (HCT). **(G)** Mean corpuscular volume (MCV). **(H)** Erythropoietin (*EPO*) mRNA levels in the kidneys. **(I)** Serum EPO protein levels detected by enzyme-linked immunosorbent assay (ELISA). **(J,K)** Serum and intestinal iron content detected by the ferrozine iron assays. **(L)** *HAMP* mRNA levels in the livers. **(M)** Serum interleukin 6 (IL-6) protein levels detected by ELISA. **(N)** Liver iron content detected by the ferrozine iron assays. **(O)** Protein levels of NcoA4 and ferritin-H in the liver detected by western blot analysis. Gapdh was used as an internal control. **(P)** Quantification of the band intensity for panel **(O)**. From panels **(D–P)**, *n* = 3–5. The analysis of variance (ANOVA) was used for statistics to evaluate the group differences. In panels **(B–N,P)**, *P < 0.05, **P < 0.01, ***P < 0.001, *Irp2*^–/–^ DMSO vs. WT DMSO; ^#^*P* < 0.05, ^##^*P* < 0.01, ^###^*P* < 0.001, ^####^*P* < 0.0001, *Irp2*^–/–^ PT-2385 vs. *Irp2*^–/–^ DMSO; ^ns^*P* > 0.05, *Irp2*^–/–^ PX-478 vs. *Irp2*^–/–^ and *Irp2*^–/–^ PT-2385 vs. *Irp2*^–/–^ DMSO.

As presented above, we only observed the upregulation of Hif2α in central nervous tissues of *Irp2*^–/–^ mice, but both inhibitors, PX-478 (5 mg/kg body weight) for Hif1α and PT-2385 (0.4 mg/kg) for Hif2α, were still injected into *Irp2*^–/–^ mice intraperitoneally every other day for 1 month, individually. During the 1 month, the weight of the mice was monitored and was found to increase normally without difference compared with the vehicle treatment, indicating the safety of the drug and its dosage (Figure 2A). In terms of behavioral performance, the latency time of *Irp2*^–/–^ mice on the rotating rod and the hanging time on the wire mesh square were significantly shorter than those of the WT mice. However, it significantly recovered by 50% in *Irp2*^–/–^ mice after administration of PT-2385, while no efficacy was observed after PX-478 treatment, which is in agreement with Hif1α levels that did not change in the CNS tissues of mutant mice (Figures 2B,C). Through inhibition of Irp2 deficiency-induced Hif2α, we confirmed the effect of PT-2385 in *Irp2*^–/–^ mice, and we also treated the WT mice with PT-2385. The rotarod and hang tests did not show a significant difference between the vehicle and PT-2385 treatment (not shown). These data proved the critical role of Hif2α in CNS of *Irp2*^–/–^ mice. Since then, our work focused on Hif2α inhibition by PT-2385.

The anemia of *Irp2*^–/–^ mice, likely, resulted from the decreased expression of TfR1 in erythroblasts and decreased bone marrow iron stores (Cooperman et al., 2005; Galy et al., 2005). Very interestingly, PT-2385 treatment corrected the anemia of *Irp2*^–/–^ mice as well, showing reversed number of RBCs, hemoglobin, and hematocrit, but not the MCV (Figures 2D–G). Then, we measured more parameters to evaluate the iron status, including the *EPO* mRNA in the kidney, serum EPO, and iron. Surprisingly, the iron status globally improved (Figures 2H–J). To understand how the PT-2385 treatment corrected the iron insufficiency anemia of *Irp2*^–/–^ mice, we assessed the iron content in the intestine and liver, *HAMP* mRNA level in the liver, and serum IL-6 to determine whether the serum iron resulted from intestinal uptake or iron release from the liver. The results showed that *HAMP* mRNA levels in the liver, serum IL-6, and iron content in the intestine and liver all reduced to be comparable with that in WT after PT-2385 treatment (Figures 2K–N), suggesting that both ways, intestinal uptake and iron release from the liver, contributed to the elevation of the serum iron. This assumption was further supported by the increased NcoA4 (Figures 2O,P), which is involved in ferritinophagy for ferritin degradation to release iron (Mancias et al., 2014).

### The Administration of PT-2385 Protects the Histological Morphology and Mitochondrial Ultrastructure in the Spinal Cord and Cerebellum of *Irp2*^–/–^ Mice

**FIGURE 3:**
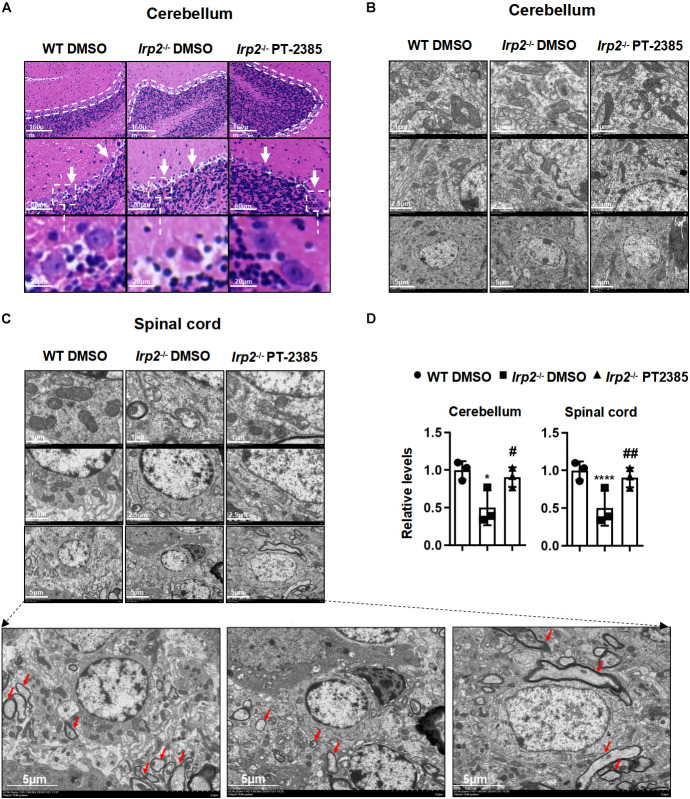
The histological morphology and mitochondrial ultrastructure in the spinal cord and cerebellum of *Irp2*^–/–^ mice are improved after PT-2385 administration. **(A)** The hematoxylin–eosin (H&E)-stained sections of the cerebellum of WT DMSO, *Irp2*^–/–^ DMSO, and *Irp2*^–/–^ PT-2385 mice. The dotted lines indicate Purkinje cell layers (top), and the arrows point to Purkinje cells (middle). The Purkinje cells framed by the dotted line are magnified four times (bottom). The scale bars are 160, 80, and 20 μm, respectively. **(B,C)** Transmission electron micrographs of the cerebellum **(B)** and spinal cord **(C)** of WT DMSO, *Irp2*^–/–^ DMSO, and *Irp2*^–/–^ PT-2385 mice. The scale bars are 1, 2.5, and 5 μm, respectively. The bottom panels are magnified images of myelin sheath and axonal degeneration. **(D)** The quantification of a normal mitochondria (relative ratio comparing with that in WT). Values represented the mean ± SD, *n* = 3. The ANOVA was used for statistics to evaluate the group differences. *P < 0.05, ****P < 0.0001, *Irp2*^–/–^ DMSO vs. WT DMSO; ^#^*P* < 0.05, ^##^*P* < 0.01, *Irp2*^–/–^ PT-2385 vs. *Irp2*^–/–^ DMSO.

Previous studies have demonstrated that misregulation of iron metabolism from loss of *Irp2* causes neuronal degeneration and mitochondrial dysmorphology (Jeong et al., 2011). The cerebellum and spinal cord are among the most severely affected tissues, so we examined them hereinafter. The H&E staining revealed that cerebellar Purkinje cells were full, intact, and tightly arranged in line in WT mice, while they were severely shrunk and/or missing in *Irp2*^–/–^ mice. However, PT-2385 treatment significantly suppressed the cerebellar Purkinje cells from degeneration or loss in *Irp2*^–/–^ mice (Figure 3A). The results from the electron microscopy showed that the density of the mitochondria in mutant cerebellum is lightened, a phenomenon which was much more severe in the spinal cord of *Irp2*^–/–^ mice than that in WT mice. More affectedly, the morphology of the mitochondria in the spinal cord of *Irp2*^–/–^ mice became swollen, vacuolated, and internal-cristae damaged. Then again, PT-2385 treatment significantly alleviated the poor presentation, including the deformed mitochondria and Wallerian and segmental demyelination (Figures 3B–D), suggesting the beneficial effect of PT-2385 against motor neurodegeneration.

### The Administration of PT-2385 Effectively Rescues the Weakened Oxidative Phosphorylation in the Cerebellum and Spinal Cord of *Irp2*^–/–^ Mice

**FIGURE 4:**
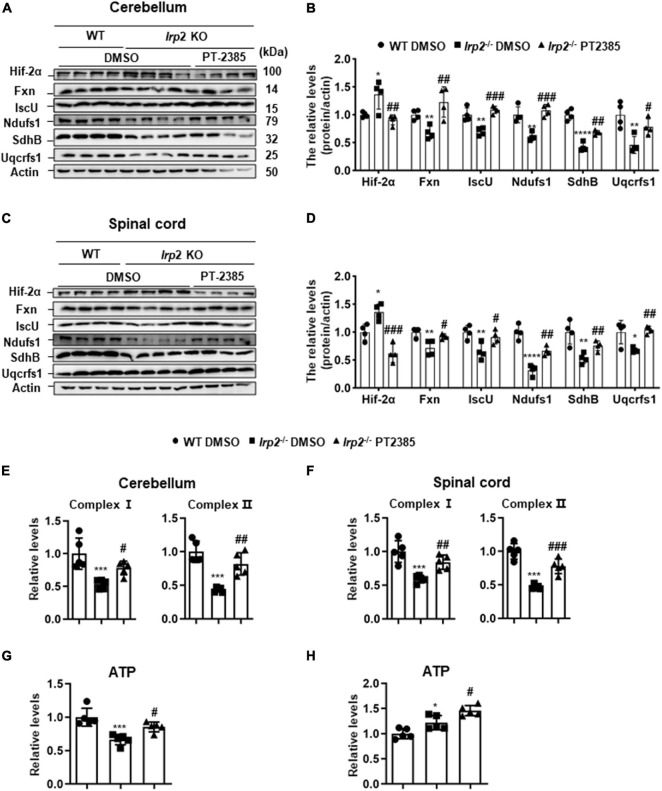
Inhibition of Hif2α by PT-2385 effectively rescues mitochondrial function in the cerebellum and spinal cord of *Irp2*^–/–^ mice. **(A)** Protein levels of Fe–S biogenesis-related genes (Fxn and IscU) and mitochondrial complex subunits (Ndufs1, SdhB, and Uqcrfs1) in the cerebellum detected by western blot analysis. Actin was used as an internal control. **(B)** Quantification of the band intensity for panel **(A)**. **(C)** The same proteins as in panel **(A)** in the spinal cord detected by western blot analysis. Actin was used as an internal control. **(D)** Quantification of the band intensity for panel **(C)**. **(E,F)** Activities of electron transport chain (ETC) complexes I and II of the cerebellums **(E)** and spinal cord **(F)**. **(G,H)** The ATP content in the cerebellum **(G)** and spinal cord **(H)**. Values represented the mean ± SD *n* = 3–5, and the ANOVA was used for statistics to evaluate the group differences. In panels **(B,D–H)**, *P < 0.05, **P < 0.01, ***P < 0.001, ****P < 0.0001, *Irp2*^–/–^ DMSO vs. WT DMSO; ^#^*P* < 0.05, ^##^*P* < 0.01, ^###^*P* < 0.001, *Irp2*^–/–^ PT-2385 vs. *Irp2*^–/–^ DMSO.

According to the results in Figure 1 and previous studies (Li et al., 2018, 2019), *Irp2* depletion induced mitochondrial dysfunction *via* reduction of Fe–S biogenesis. We evaluated the levels of Fxn and IscU and of complex subunits, Ndufs1, SdhB, and Uqcrfs1, in the cerebellum and spinal cord tissues of *Irp2*^–/–^ mice. Again, Hif2α was found increased, and the expression of mitochondrial proteins, including Fxn, IscU, and complex subunits, was decreased in *Irp2*^–/–^ mice compared with WT. However, PT-2385 treatment inhibited all these biochemical changes compared with *Irp2*^–/–^ mice (Figures 4A–D). In line with these results, the activities of mitochondrial complexes I and II were also significantly restored in both tissues (Figures 4E,F). The exception is the coupled ETC product ATP. In the cerebellum, ATP content was lower in *Irp2*^–/–^ mice than that in WT mice, and PT-2385 administration increased it, which is correlated with the ETC activities (Figure 4G). However, in the spinal cord, ATP content increased significantly in *Irp2*^–/–^ mice (Figure 4H), which is consistent with the previous studies (Li et al., 2018, 2019) in MEFs, though the ETC-related proteins and enzymatic activities were lower in *Irp2*^–/–^ mice than those in WT mice (Figures 4C,D,F). The reason will be discussed further in section “Discussion.” Very surprisingly, compared with *Irp2*^–/–^ mice, more ATP was produced after PT-2385 administration (Figure 4H). Overall, the inhibition of Hif2α by PT-2385 rescues the weakened OXPHOS in *Irp2*^–/–^ mice to provide more ATP to fulfill the energy need.

### The Administration of PT-2385 Attenuates Enhanced Glycolysis in the Cerebellum and Spinal Cord of *Irp2*^–/–^ Mice

**FIGURE 5:**
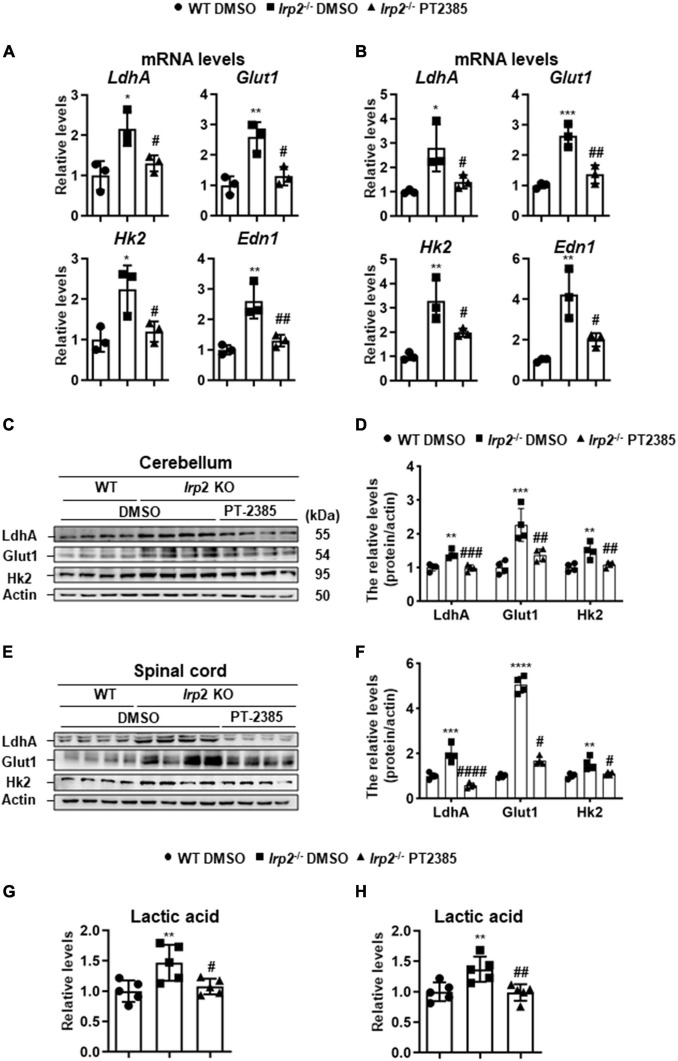
Inhibition of Hif2α by PT-2385 attenuates the enhanced glycolysis in the cerebellum and spinal cord of *Irp2*^–/–^ mice. **(A,B)** The mRNA levels of Hif-target genes *LdhA*, *Glut1*, *Hk2*, and *Edn1* in the cerebellum **(A)** and spinal cord **(B)**, detected by qPCR. **(C)** Glycolytic pathway-related proteins (Glut1, Hk2, and LdhA) in the cerebellum detected by western blot analysis. Actin was used as an internal control. **(D)** Quantification of the band intensity for panel **(C)**. **(E)** The same proteins as in panel **(C)** in the spinal cord detected by western blot analysis. Actin was used as an internal control. **(F)** Quantification of the band intensity for panel **(E)**. **(G,H)** The lactic acid levels in the cerebellum **(G)** and spinal cord **(H)**. Values represented the mean ± SD, *n* = 3–5. The ANOVA was used for statistics to evaluate the group differences. In panels **(A,B,D,F–H)**, *P < 0.05, **P < 0.01, ***P < 0.001, ****P < 0.0001, *Irp2*^–/–^ DMSO vs. WT DMSO; ^#^*P* < 0.05, ^##^*P* < 0.01, ^###^*P* < 0.001, ^####^*P* < 0.0001, *Irp2*^–/–^ PT-2385 vs. *Irp2*^–/–^ DMSO.

Though Hif1α was not found to be upregulated *in vivo*, we observed the enhanced glycolysis-related gene expression (Figure 1B), which is similar to the previous results in *Irp2*^–/–^ MEFs (Li et al., 2019). We still used the cerebellum and spinal cord tissues to check the effect of PT-2385 on the expression of *LdhA*, *Glut1*, *Hk2*, and *Edn1*, genes that are considered to be the members of Hif regulon. The results showed that the expression of these genes increased by 100–150% in the cerebellum and by 170–300% in the spinal cord of *Irp2*^–/–^ mice and reduced to the WT levels after PT-2385 treatment (Figures 5A,B), confirming the action of PT-2385 on Hif2α and the regulon relationship of Hif2α to the tested genes. The protein levels of glycolysis-related genes including LdhA, Glut1, and Hk2 were also significantly reduced after PT-2385 treatment (Figures 5C–F). Among them, Glut1 showed extraordinary change in the spinal cord. Accordingly, the lactic acid levels were significantly higher in both the cerebellum and spinal cord of *Irp2*^–/–^ mice than those in WT, and PT-2385 treatment significantly lowered the levels in both tissues of *Irp2*^–/–^ mice (Figures 5G,H). Comparing the two tissues, the cerebellum and spinal cord, the upregulated Hif-targeted genes seemingly responded stronger in the spinal cord than in the cerebellum since their mRNA levels were more elevated in the spinal cord (Figure 5B) than in the cerebellum (Figure 5A), particularly for *Hk2* and *Edn1*, after Irp2 depletion. The protein levels of LdhA and Glut1 in the spinal cord increased about twofold and fivefold, respectively (Figures 5E,F), vs. 1.3- and 2-fold in the cerebellum (Figures 5C,D) after Irp2 depletion. The results suggest that the spinal cord might suffer more from the upregulated Hif2α and active glycolysis.

## Discussion

In this study, we found that Irp2 ablation induced Hif2α upregulation associated with the metabolism switch from OXPHOS to glycolysis *in vivo*. The protective effect of Hif2 inhibitor PT-2385 suggested that Hif2α could be a potential target therapeutically in the treatment of IRP2 mutant-caused neurodegenerative syndrome.

**FIGURE 6:**
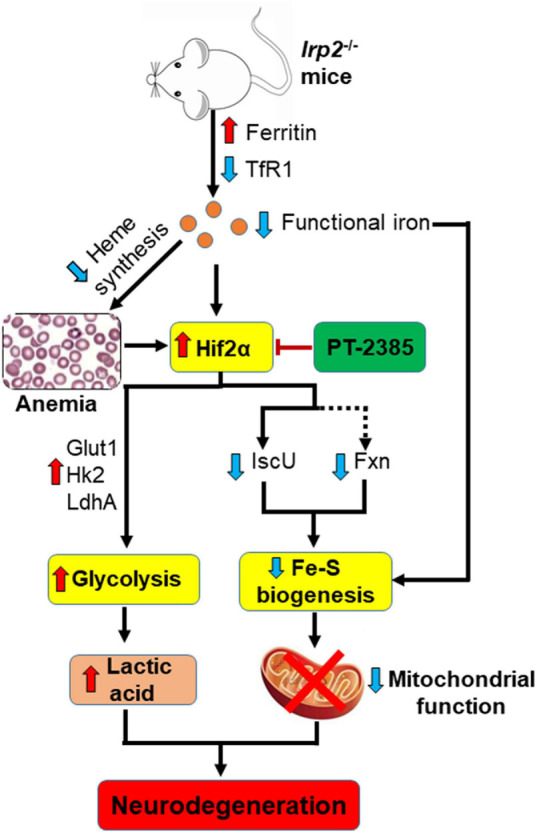
A working model to illustrate the potential target, Hif2α, in the therapeutic application of Irp2 loss-triggered neurodegenerative syndrome. *Irp2* depletion causes the deficiency of cellular functional iron by decreased transferrin receptor and increased ferritin, which compromise the heme biosynthesis and finally trigger microcytic anemia in mice. Both the iron deficiency and anemia/hypoxia upregulate Hif2α expression *in vivo*. The elevated Hif2α upregulates glycolytic pathway-related genes to enhance glycolysis, while downregulates iron–sulfur cluster biogenesis-related genes, Fxn and IscU, to weaken mitochondrial function. After the inhibition of Hif2α by PT-2385, the energy metabolism is shifted from glycolysis to oxidative phosphorylation in *Irp2*^–/–^ mice and neurodegenerative symptoms are alleviated.

Irp2 depletion causes the deficiency of cellular functional iron by decreasing transferrin receptor and increasing ferritin expression (Jeong et al., 2011), which compromise the heme biosynthesis and finally trigger microcytic anemia in mice (Cooperman et al., 2005; Galy et al., 2005) and in humans (Cooper et al., 2019; Costain et al., 2019). Both the functional iron deficiency and anemia-induced low oxygen tension/hypoxia could upregulate Hif2α expression *in vivo*. In our previous study, we did find that Irp2 ablation induced the increase of Hif1α and Hif2α in *Irp2*^–/–^ MEFs (Li et al., 2019). However, we did not find an increase of Hif1α, but of Hif2α, in *Irp2*^–/–^ mice. Inhibition of Hif2α by PT-2385 greatly alleviated the progress of neurodegeneration accompanied with the enhancement of mitochondrial Fe–S biogenesis and suppression of glycolytic pathway-related proteins. In agreement with previous studies *in vitro* (Li et al., 2018, 2019), the loss of *Irp2* in mice downregulated the expression of Fxn and IscU, two important core components in Fe–S biogenesis machinery. Increase of Fxn and IscU in *Irp2*^–/–^ cells, either by overexpression of the two genes (Li et al., 2018) or by inhibition of Hif2α (Li et al., 2019; this study), augments the mitochondrial complex activities and ATP content. At the same time, inhibition of Hif2α weakened glycolysis to avoid the toxicity of high levels of lactic acid through suppression of *LdhA*, *Glut1*, and *Hk2*. Therefore, PT-2385 administration dramatically protected from the progressive neurodegeneration as modeled in Figure 6.

Interestingly, PT-2385 administration also improved overall blood parameters from anemia. However, Hif2 is very important for erythropoiesis by regulating EPO production and for iron uptake in the small intestine by regulating DMT1, FPN1, and Dcytb. Surprisingly, we found that the lifted iron content in the liver of *Irp2* mutant reduced and the increased ferritin dropped back to WT levels. More profoundly, we found that NcoA4 expression increased in the liver after Hif2 inhibition, suggesting the important role of NcoA4 for iron release from ferritin, very likely, through ferritinophagy.

Both Hif1α and Hif2α are regulated by oxygen and iron (Majmundar et al., 2010; Prabhakar and Semenza, 2012). Both the functional iron deficiency and induced anemia could upregulate Hif1α expression as well. However, we did not see similar responses of Hif1α with Hif2α in this study. We found that elevated Hif2α alone, not together with Hif1α, contributes to the switch of energy metabolism from OXPHOS to glycolysis. The rationale for Hif2α to regulate IscU has been demonstrated in which IscU is a member of the miR-210 regulon (Chan et al., 2009) and the promoter of miR-210 contains a HRE for Hif1/2 binding (Kulshreshtha et al., 2007). Therefore, downregulation of IscU may be explained to be through the miR-210–Hif2 axis in *Irp2*^–/–^ mice. The accompanied co-regulation of Fxn with IscU was often observed (Ferecatu et al., 2018; Li et al., 2018, 2019), and the detailed regulation mechanism of Hif2α on Fxn remains to be explored. If mouse Fxn was regulated by Hif2α as reported (Oktay et al., 2007), it would be expected that Fxn expression should be increased. Indeed, Irp2 depletion induced the downregulation of Fxn, the expression of which was reversed after inhibition of Hif2α by PT-2385. The co-regulation of Fxn and IscU could be the key to response to PT-2385 treatment since the interaction of Fxn with IscU is important to facilitate Fe–S biogenesis (Fox et al., 2019; Gervason et al., 2019) to cure mitochondrial dysfunction. A similar work has been reported in which neuronal Hif1α and Hif2α deficiency improves neuronal survival and sensorimotor function in the early acute phase after ischemic stroke (Barteczek et al., 2017).

Though the 5′-UTR of *Hif2*α mRNA contains an IRE element for Irp1 binding (Sanchez et al., 2007; Barteczek et al., 2017), Irp1–IRE binding activity keeps constant in Irp2-depleted tissues (Zumbrennen-Bullough et al., 2014). Hif2α is, likely, upregulated by iron deficiency and anemia-induced hypoxia in *Irp2*^–/–^ mice as discussed above. Another regulation is also possible as XIAP and Ubc13-dependent Lys63-linked polyubiquitination promotes Hif1α nuclear retention leading to an increase in the expression of Hif1-responsive genes (Park et al., 2017). We do not know if a similar pathway exists for Hif2α stabilization. Interestingly, *Irp1*^–/–^-induced elevated Hif2α upregulates EPO, causing the mice to develop polycythemia and pulmonary hypertension (Anderson et al., 2013; Ghosh et al., 2013; Wilkinson and Pantopoulos, 2013). However, the upregulated EPO expression in *Irp2*^–/–^ mice is probably invalid due to the iron limit in the bone marrow (Cooperman et al., 2005). Moreover, the increased Hif2α endowed glycolysis-related genes, such as *LdhA*, *Glut1*, and *Hk2*, in the cerebellum and spinal cord of *Irp2*^–/–^ mice. Therefore, inhibition of Hif2 by PT-2385 did not only increase the expression of Fxn and IscU to strengthen mitochondrial function but also decrease the expression of *LdhA*, *Glut1*, and *Hk2* to weaken glycolysis to avoid the toxicity of high level of lactic acid. Although according to the astrocyte–neuron lactate shuttle hypothesis, lactic acid can be used as an energy metabolism substrate for neurons, and Irp2 deficiency-induced mitochondrial dysfunction is insufficient to meet the energy needs in neurons. Intriguingly, elevated lactic acid was observed more in the spinal cord than in the cerebellum, which is consistent with more severity in motor than in other behaviors (Jeong et al., 2011). The enhanced glycolysis could be the reason why the ATP production was slightly, but significantly, more in the spinal cord than in the cerebellum after *Irp2* ablation.

The patient with the absence of IRP2 shows functional iron deficiency and mitochondrial dysfunction that emulate *Irp2*^–/–^ mice (Costain et al., 2019). The complete loss of IRP2 in patient-derived lymphoblasts also induces the decreased expression of complex subunits and activities of mitochondrial complexes I and II (Costain et al., 2019), although the expression levels of Hifs, Fxn, IscU, and glycolytic pathway-related proteins are not detected. We expect that Hif2α is upregulated in the tissues of the CNS in patients as we observed in *Irp2*^–/–^ mice, and the inhibition of Hif2 may be a therapeutic option for the IRP2-loss patients. On the other hand, genetical knockdown of *Hif2*α in *Irp2*^–/–^ mice needs to be carried out to firmly validate the effects of Hif2 inhibition. The mechanism of action of PT-2385 is thought to selectively antagonize Hif2 heterodimerization, and DNA-binding activity and has no effect on Hif1 and no significant off-target activity (Yu et al., 2017).

## Conclusion

In summary, we have demonstrated that *Irp2* ablation induces the expression of Hif2α, not Hif1α, in the tissues of CNS of *Irp2*^–/–^ mice, and Hif2 inhibitor PT-2385 dramatically protects the mice from the neurodegenerative disorder accompanied with the energy metabolism shift from glycolysis to OXPHOS, indicating that Hif2α is a potential target for neurodegenerative syndrome caused by loss of IRP2.

## Data Availability Statement

The original contributions presented in the study are included in the article/Supplementary Material, further inquiries can be directed to the corresponding authors.

## Ethics Statement

The animal study was reviewed and approved by the Animal Experimentation Administration of Nanjing University.

## Author Contributions

JS, EH, TQ, and KL conceptualized the study. JS conducted the formal analysis and performed the visualization. TQ and KL acquired the funding. JS, LX, YXL, WD, YTL, HZ, and TX conducted the investigation. JS, LX, JC, TX, and KL performed the methodology. JS and KL were involved in project administration, wrote the original draft, and reviewed and edited the manuscript. YC gathered the resources. JS, YXL, and YTL performed the validation. All authors contributed to the article and approved the submitted version.

## Conflict of Interest

The authors declare that the research was conducted in the absence of any commercial or financial relationships that could be construed as a potential conflict of interest.

## Publisher’s Note

All claims expressed in this article are solely those of the authors and do not necessarily represent those of their affiliated organizations, or those of the publisher, the editors and the reviewers. Any product that may be evaluated in this article, or claim that may be made by its manufacturer, is not guaranteed or endorsed by the publisher.
