# Correction: Protective effects of Hif2 inhibitor PT-2385 on a neurological disorder induced by deficiency of Irp2

**DOI:** 10.3389/fnins.2025.1667076

**Published:** 2025-08-29

**Authors:** Jiaqi Shen, Li Xu, Yuxuan Li, Weichen Dong, Jing Cai, Yutong Liu, Hongting Zhao, Tianze Xu, Esther Meyron Holtz, Yanzhong Chang, Tong Qiao, Kuanyu Li

**Affiliations:** ^1^Jiangsu Key Laboratory of Molecular Medicine, Medical School of Nanjing University, Nanjing, China; ^2^Department of Neurology, The Affiliated Jinling Hospital of Nanjing University Medical School, Nanjing, China; ^3^Department of Vascular Surgery, The Affiliated Drum Tower Hospital of Nanjing University Medical School, Nanjing, China; ^4^The Laboratory of Molecular Nutrition, Faculty of Biotechnology and Food Engineering, Technion – Israel Institute of Technology, Haifa, Israel; ^5^College of Life Science, Hebei Normal University, Shijiazhuang, China

**Keywords:** iron regulatory protein 2, hypoxia inducible factor 2α, glycolysis, oxidative phosphorylation, iron sulfur cluster, neurodegeneration

There was a mistake in Figure 3 as published. One image was wrongly used in Figure 3B (left middle one), which is the same as in Figure 3C (right middle one). The corrected Figure 3 appears below.

**Figure 3:**
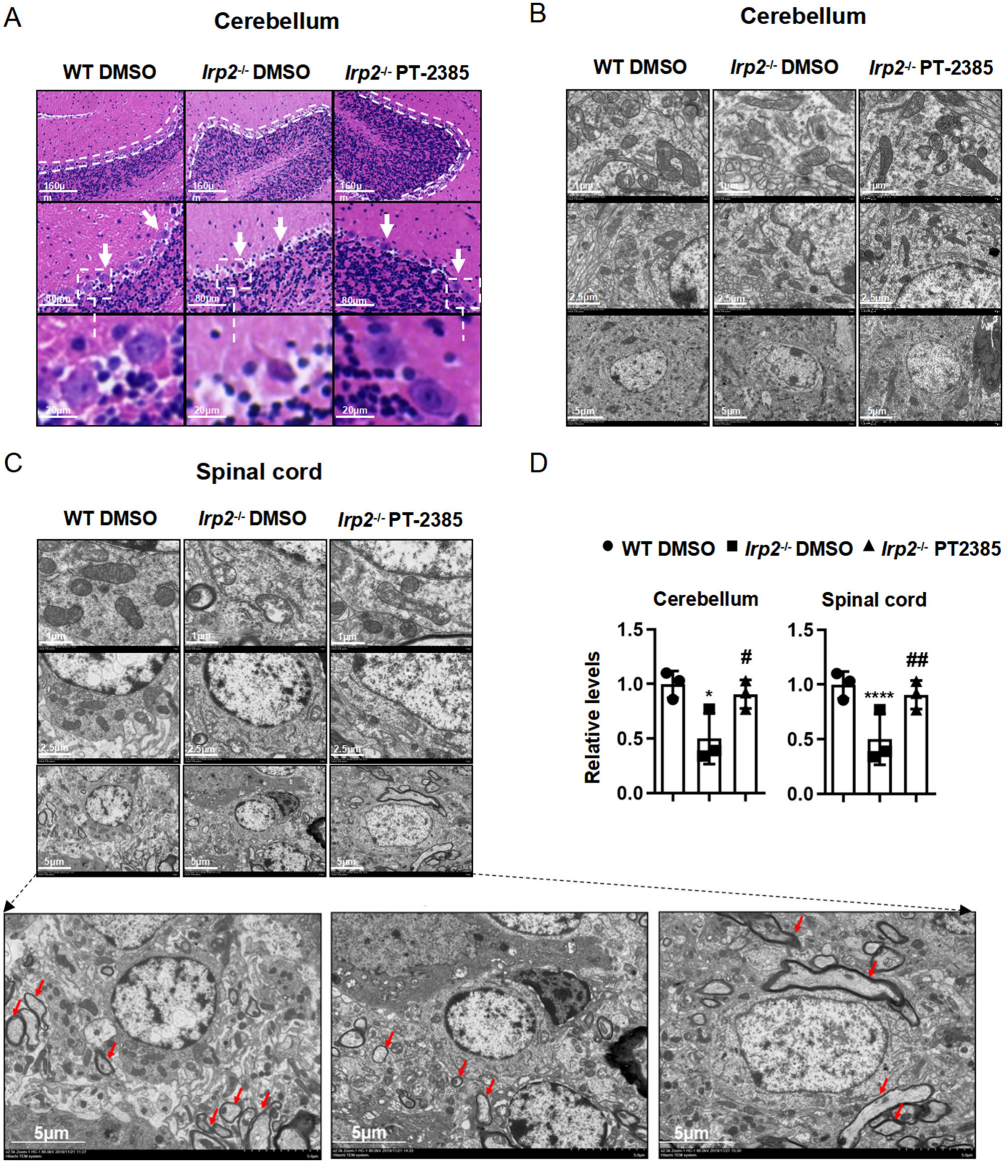
The histological morphology and mitochondrial ultrastructure in the spinal cord and cerebellum of *Irp*2^−/−^ mice are improved after PT-2385 administration. **(A)** The hematoxylin–eosin (H&E)-stained sections of the cerebellum of WT DMSO, *Irp*2^−/−^ DMSO, and *Irp*2^−/−^ PT-2385 mice. The dotted lines indicate Purkinje cell layers (top), and the arrows point to Purkinje cells (middle). The Purkinje cells framed by the dotted line are magnified four times (bottom). The scale bars are 160, 80, and 20 μm, respectively. **(B, C)** Transmission electron micrographs of the cerebellum **(B)** and spinal cord **(C)** of WT DMSO, *Irp*2^−/−^ DMSO, and *Irp*2^−/−^ PT-2385 mice. The scale bars are 1, 2.5, and 5 μm, respectively. The bottom panels are magnified images of myelin sheath and axonal degeneration. **(D)** The quantification of a normal mitochondria (relative ratio comparing with that in WT). Values represented the mean ± SD, *n* = 3. The ANOVA was used for statistics to evaluate the group differences. ^*^P < 0.05, ^****^P < 0.0001, *Irp*2^−/−^ DMSO vs. WT DMSO; ^#^*P* < 0.05, ^##^*P* < 0.01, *Irp*2^−/−^ PT-2385 vs. *Irp*2^−/−^ DMSO.

The original version of this article has been updated.

